# Lightweight Reinforcement Learning for Priority-Aware Spectrum Management in Vehicular IoT Networks

**DOI:** 10.3390/s25216777

**Published:** 2025-11-05

**Authors:** Adeel Iqbal, Ali Nauman, Tahir Khurshaid

**Affiliations:** 1 School of Computer Science and Engineering, Yeungnam University, Gyeongsan-si 38541, Republic of Korea; adeeliqbal@yu.ac.kr; 2 Department of Electrical Engineering, Yeungnam University, Gyeongsan-si 38541, Republic of Korea

**Keywords:** V-IoT, QoS, reinforcement learning, Markov Decision Process, 5G, IoT, priority-aware spectrum management, spectrum access, resource allocation

## Abstract

The Vehicular Internet of Things (V-IoT) has emerged as a cornerstone of next-generation intelligent transportation systems (ITSs), enabling applications ranging from safety-critical collision avoidance and cooperative awareness to infotainment and fleet management. These heterogeneous services impose stringent quality-of-service (QoS) demands for latency, reliability, and fairness while competing for limited and dynamically varying spectrum resources. Conventional schedulers, such as round-robin or static priority queues, lack adaptability, whereas deep reinforcement learning (DRL) solutions, though powerful, remain computationally intensive and unsuitable for real-time roadside unit (RSU) deployment. This paper proposes a lightweight and interpretable reinforcement learning (RL)-based spectrum management framework for Vehicular Internet of Things (V-IoT) networks. Two enhanced Q-Learning variants are introduced: a Value-Prioritized Action Double Q-Learning with Constraints (VPADQ-C) algorithm that enforces reliability and blocking constraints through a Constrained Markov Decision Process (CMDP) with online primal–dual optimization, and a contextual Q-Learning with Upper Confidence Bound (Q-UCB) method that integrates uncertainty-aware exploration and a Success-Rate Prior (SRP) to accelerate convergence. A Risk-Aware Heuristic baseline is also designed as a transparent, low-complexity benchmark to illustrate the interpretability–performance trade-off between rule-based and learning-driven approaches. A comprehensive simulation framework incorporating heterogeneous traffic classes, physical-layer fading, and energy-consumption dynamics is developed to evaluate throughput, delay, blocking probability, fairness, and energy efficiency. The results demonstrate that the proposed methods consistently outperform conventional Q-Learning and Double Q-Learning methods. VPADQ-C achieves the highest energy efficiency (≈$8.425\times 10^{7}$ bits/J) and reduces interruption probability by over 60%, while Q-UCB achieves the fastest convergence (within ≈190 episodes), lowest blocking probability (≈0.0135), and lowest mean delay (≈0.351 ms). Both schemes maintain fairness near 0.364, preserve throughput around 28 Mbps, and exhibit sublinear training-time scaling with O(1) per-update complexity and $O(N^{2})$ overall runtime growth. Scalability analysis confirms that the proposed frameworks sustain URLLC-grade latency (<0.2 ms) and reliability under dense vehicular loads, validating their suitability for real-time, large-scale V-IoT deployments.

## 1. Introduction

The rapid development of autonomous and connected cars has turned the Vehicular Internet of Things (V-IoT) into a key technology for future intelligent transportation systems (ITSs). Applications such as safety-related collision avoidance, cooperative awareness, infotainment, and fleet management need smooth connectivity with strict requirements for latency, reliability, and scalability. However, the wireless spectrum remains a scarce resource, shared across heterogeneous vehicular uses with mixed quality-of-service (QoS) priorities. Addressing these disparate requirements requires spectrum access structures that are efficient as well as adaptive to rapidly evolving vehicular scenarios [1].

**Figure 1:**
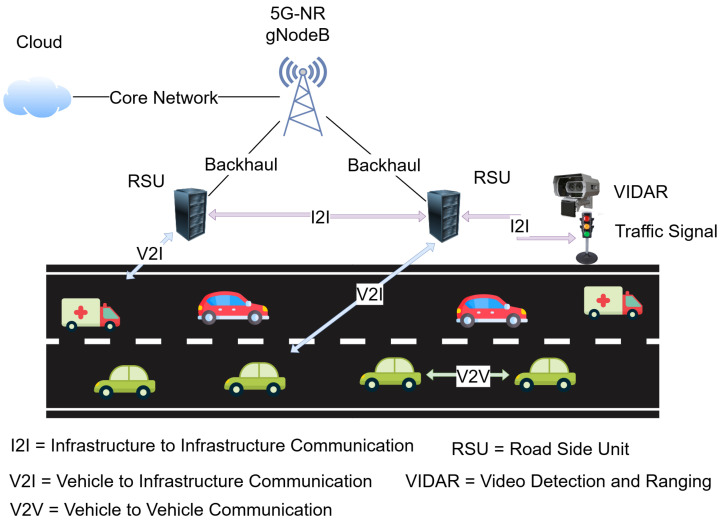
Overview of vehicular communication paradigms.

Three paradigms for vehicular communication are depicted in Figure 1. Vehicle-to-Vehicle (V2V) links allow peer-to-peer interactions among proximal vehicles, supporting time-critical messages such as collision avoidance and cooperative awareness. Decentralized groups provide very low latency but are constrained by interference in the spectrum and the highly dynamic vehicular graph. Vehicle-to-Infrastructure (V2I) communication extends connectivity to roadside units (RSUs) and base stations, enabling centralized resource management, traffic signal control, and access to cloud-based services. V2I is particularly pertinent, as it is the interface wherein multiple vehicles contend for spectrum under varying traffic loads. Infrastructure-to-Infrastructure (I2I) communication contributes to network robustness by interconnecting RSUs and base stations through backhaul links, allowing inter-cell coordination, spectrum sharing, and handoff assistance. While the three modes all contribute to the V-IoT ecosystem, the most stringent and impactful spectrum access decisions occur in the V2I domain. To this end, this research focuses on RL-based spectrum management at the RSU level for efficient, reliable, and fair channel assignment across safety-critical, control, and infotainment traffic classes in dense vehicular deployments.

Classical resource-allocation policies such as round-robin scheduling, proportional fair access, and static priority queues are insufficient in such cases. Though deterministic, they are not responsive enough to adapt to the stochastic arrival process of vehicular traffic, time-varying channel conditions, and diverse QoS requirements. In recent years, RL techniques have been investigated as a means to assist adaptive spectrum management by learning about the environment through interaction. Deep RL methods have proven to be promising; however, their prohibitively expensive training complexity, large memory needs, and high inference latency make them inapplicable for real-time applications on RSUs or vehicular edge units. Such practical constraints motivate the formulation of lightweight, interpretable learning models that retain the flexibility of RL but are computationally feasible for the V-IoT.

The limitations of DRL in automotive environments arise not only from hardware but also from operational and temporal dynamics. Although RSUs typically enjoy stable power supplies and good computing resources, the real challenge lies in remaining online and adaptive under highly dynamic network conditions. DRL agents trained offline often suffer poor performance when spectrum availability, vehicular traffic, or interference patterns deviate from the training data distribution. Continuous online retraining of deep models involves significant latencies, memory, and signaling overhead between vehicular agents and RSUs, which makes them unsuitable for supporting URLLC applications. Additionally, DRL-based designs involve significant communication overhead in synchronized policy distribution and parameter sharing between vehicular agents and RSUs, limiting their scalability in dense V-IoT environments. Apart from this, distributed DRL introduces synchronization delay and additional bandwidth use for parameter updates between nodes [2]. On the other hand, light RL strategies provide deterministic updates, faster convergence rates, and higher interpretability, all of which are essential for safety-critical vehicular operations with clear decision-making rules. These features inspire the design of low-computational-expense, interpretable learning mechanisms that retain the flexibility of RL without compromising feasibility for real-time deployment in V-IoT systems [3]. Apart from schedulers based on learning, heuristic baselines are also beneficial due to their explainability and low computational overhead for purposes of benchmarking. In this manuscript, a Risk-Aware Heuristic is introduced as a non-learning baseline that exploits queue length, signal quality, and traffic priority to make cautious scheduling decisions under uncertainty. This establishes an interpretable baseline against which the benefits of adaptive RL techniques can be compared objectively.

Traditional Q-Learning provides off-policy value iteration with convergence guarantees subject to suitable sampling and step-size conditions [4]. However, the max operator induces overestimation bias in noisy settings, and DoubleQ prevents this with decoupled action choice and evaluation [5]. Exploration is still of utmost importance; Upper Confidence Bound (UCB)-style optimism gives tight regret for bandits [6] and, when added as a bonus to Q-Learning, gives provably sample-efficient exploration in episodic and infinite-horizon tabular MDPs [7]. RL has been extensively utilized in vehicular communications and dynamic spectrum sharing to acquire sub-band and power allocation under time-varying interference, with great performance achieved by multi-agent and deep RL variants in decentralized V2V/V2X settings [8,9,10]. Recent research has also explored GNN- and federated-RL-based schedulers for scalability and privacy [11]. Fairness is generally represented by the Jain index, which is population-size independent and bounded [12]. Energy efficiency (bits/Joule) has become a salient objective, along with QoS, for modern wireless and IoT applications, and DRL methods have focused more on joint rate–latency–energy trade-offs [9,13,14]. To address this limitation, we focus our work on tabular Q-Learning and its state-of-the-art variants as spectrum management methods for the V-IoT. Q-Learning provides an intuitive and efficient framework for adaptive decision-making by mapping observed states to optimal channel access actions.

We improve this baseline in two ways. First, we develop a Value-Prioritized Action Double Q-Learning with Constraints (VPADQ-C) algorithm, which unites double estimation with priority-aware updates to address value overestimation while offering higher scheduling priority to key traffic. Second, we introduce a Q-Learning with Upper Confidence Bound (Q-UCB) extension, which includes statistical confidence rewards in exploration to speed up convergence and improve responsiveness under evolving vehicular contexts. By comparing these strategies rigorously to the vanilla Q-Learning baseline, we demonstrate that light RL can achieve unprecedented boosts in throughput, blocking probability, fairness, and energy efficiency without deep RL’s computational cost. The key contributions of this paper are as follows:

- We propose a priority-aware spectrum management system for the V-IoT, inspired by safety-constrained lightweight reinforcement learning and interpretability.
- We design two enhanced Q-Learning methods, VPADQ-C and Q-UCB, which co-embed a Constrained Markov Decision Process (CMDP)-foundation-based safety controller, contextual uncertainty-based explorations, and a Success-Rate Prior (SRP) to provide improved stability and adaptability.
- We introduce a Risk-Aware Heuristic baseline for a fair comparison of interpretability and robustness, such that rule-based and learning-based scheduling methods have a level playing field.
- We develop an end-to-end simulation and testing platform considering physical-layer energy, throughput, and fairness under realistic vehicular mobility and channel conditions.
- We perform exhaustive scalability and complexity analyses, demonstrating that all of the suggested techniques possess robust performance that is maintained as the number of vehicles increases, with per-update complexity of O(1) and aggregate runtime scaling of $O(N^{2})$.

The rest of this manuscript is organized as follows. Section 2 provides an overview of recent advances in V-IoT networks. Section 3 elaborates on the system model. Section 4 presents the designed framework and learning approaches. Section 5 outlines the simulation environment and parameters. Section 6 discusses the results and makes key observations. Lastly, Section 7 concludes this research.

## 2. Literature Review

RL has been widely investigated to control the spectrum for vehicular networks, particularly in the V2I setting. The authors of [15] introduced a multi-agent RL framework under which V2V links learn to reclaim V2I spectrum for reuse by employing distributed deep Q-Learning, which optimizes V2I capacity without compromising V2V reliability. This was further enhanced in [16] using an actor–critic approach for concurrent mode selection, channel assignment, and power control with a URLLC guarantee. The authors of [17] also described the potential of decentralized RL, with recurrent dueling DQNs used to enable vehicles to access spectrum independently without global channel state information, achieving almost centralized performance. The study in [18] applied federated learning and multi-agent RL for scalability and privacy purposes, with vehicles able to collectively learn spectrum access policies with data locality. Complementing these communication-centric approaches, the authors of [19] proposed a Lyapunov-guided deep RL approach for concurrent spectrum and computing resource allocation, ensuring queue stability and URLLC-level reliability. Although DRL-based works like these achieved good performance, their deep neural models resulted in huge training complexity and energy consumption due to frequent synchronization between distributed nodes. Such overhead limits their real-time applicability within RSU-assisted vehicular systems, where decision delay should be maintained on the order of milliseconds.

Beyond applied studies, theoretical RL foundations are directly relevant to spectrum management. Classical Q-Learning provides off-policy value iteration with convergence guarantees under suitable sampling and step-size conditions [4]. However, the max operator induces overestimation bias in noisy settings, which DoubleQ mitigates through decoupled action selection and evaluation [5]. Exploration remains central; UCB-style optimism offers tight regret bounds in bandit problems [6] and, when integrated into Q-Learning, yields provable sample efficiency in both episodic and infinite-horizon tabular MDPs [7]. RL has been extensively applied to dynamic spectrum sharing and vehicular communications to adapt sub-band and power allocation under time-varying interference, with deep and multi-agent variants showing strong performance in decentralized V2V/V2X settings [8,9,10]. Recent advances have also explored graph neural network-assisted and federated RL schedulers for scalability and privacy [11]. In parallel, lightweight RL frameworks such as model-free Q-learning with adaptive exploration, actor–critic with eligibility traces, and energy-aware tabular schedulers have been investigated for low-latency IoT applications [2,20]. These methods demonstrate that shallow, interpretable learning architectures can retain adaptivity while substantially reducing computational and communication overhead.

Fairness is often captured using the Jain index [12], while energy efficiency (bits/Joule) has become a parallel objective alongside QoS in modern wireless and IoT systems, with DRL methods increasingly targeting joint rate–latency–energy trade-offs [9,13,14]. While many prior works have addressed sidelink (PC5) resource allocation for V2V or hybrid V2V/V2I scenarios, the present study focuses on Uu-based V2I communications, in which an RSU (gNB-type) performs centralized uplink scheduling over licensed spectrum. This distinction changes the decision space: instead of distributed vehicle-side coordination, the RSU coordinates uplink transmissions from multiple IoT-enabled vehicles with heterogeneous QoS priorities. In support of this setting, NR-V2X offers dedicated uplink mechanisms such as configured grants on the Uu interface [21], and recent works have begun to examine vehicular uplink scheduling [22] and RL-based uplink schedulers in cellular/IoT environments [23].

These studies collectively establish RL as a promising tool for adaptive spectrum management in V2I networks. However, most existing solutions emphasize deep or distributed RL architectures, which remain computationally heavy and difficult to interpret for real-time RSU deployment. Only a few works have explored lightweight or tabular models capable of offering fast convergence, low memory cost, and explainable decision-making under dynamic vehicular traffic. This gap motivates the present work, which investigates enhanced Q-Learning variants tailored for V2I spectrum management, aiming to balance scalability, reliability, and real-time feasibility. Table 1 summarizes the key related studies.

## 3. System Model

**Figure 2:**
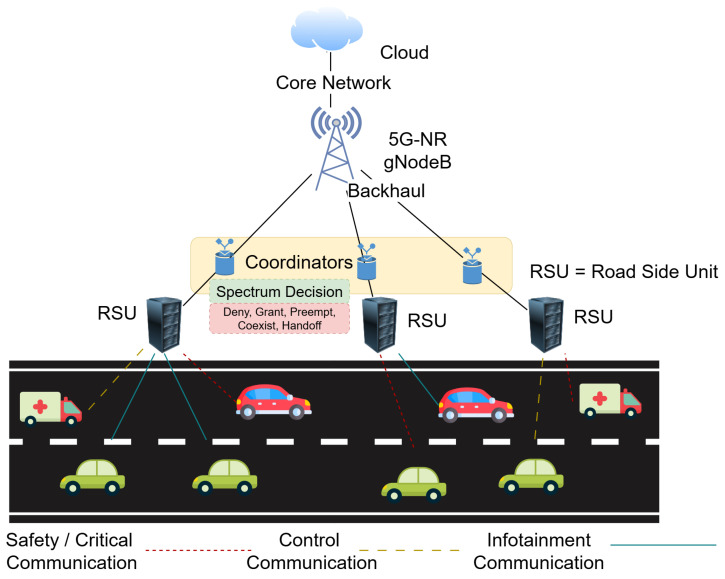
System model of V-IoT where an RSU applies RL-based spectrum management across safety, control, and infotainment traffic classes.

We consider a V-IoT environment in which an RSU manages spectrum access for multiple vehicles contending for uplink transmission opportunities. The RSU coordinates channel allocation in discrete time slots of fixed duration $T_{s}$. The available spectrum is divided into *C* orthogonal channels, each with bandwidth $B_{c}$, which can be dynamically assigned to vehicular devices. The system integrates heterogeneous traffic, physical-layer dynamics, and RL-driven spectrum access decisions. Figure 2 illustrates the overall architecture.

Vehicles generate traffic belonging to three classes: *safety-critical*, *control*, and *infotainment*. Safety-critical messages (e.g., collision alerts) require ultra-low latency and high reliability. Control traffic, such as telemetry or coordination updates, demands moderate latency and reliability, whereas infotainment data are delay-tolerant and can tolerate retransmissions. All incoming packets are queued at the RSU, which schedules transmissions while satisfying the heterogeneous QoS demands of these traffic types.

### 3.1. Network Abstraction

We consider a single-RSU cell; inter-RSU interference is modeled using a reuse-1 assumption. Although the nominal bandwidth is 100 MHz, only an *effective* bandwidth $B_{\mathrm{eff}}<100$ MHz is available after accounting for control channels (PUCCH/PDCCH/ PRACH), guard bands, and safety beacons. The MAC layer is expressed in Equation (1) as

(1) $$C=\left\lfloor \frac{B_{\mathrm{eff}}}{B_{c}}\right\rfloor$$

coarse-grained orthogonal channels (PRB-group–level units) of width $B_{c}$, which the RSU schedules every slot. This abstraction emphasizes centralized uplink coordination under heterogeneous QoS priorities rather than raw LTE/NR RB accounting.

### 3.2. Communication Interface

The system follows the V2I model on the cellular Uu interface, with IoT-supportive vehicles as user equipment (UE) transmitting to an RSU as a gNB-type edge controller. Centralized scheduling and spectrum assignment are performed by the RSU, which is aligned with practical 5G/6G vehicular deployments. While V2V or sidelink (PC5) communication is not modeled, the framework captures the key uplink dynamics of vehicular transmissions in the broader V-IoT setting.

### 3.3. Channel Model

The complex baseband channel coefficient between the RSU and the *i*-th vehicle is expressed in Equation (2) as

(2) $$h_{i}=\sqrt{G_{0}\;d_{i}^{-\alpha }\;10^{-\psi_{i}/10}}\;g_{i},$$

where $G_{0}$ denotes the reference path gain at unit distance (free-space component), $d_{i}$ is the RSU–vehicle separation, α is the path-loss exponent, $\psi_{i}∼N\left(0,\sigma_{\mathrm{sh}}^{2}\right)$ represents the log-normal shadowing term in dB, and $g_{i}∼\mathrm{CN}\left(0,1\right)$ is the small-scale Rayleigh fading coefficient. Hence, $|h_{i}{|}^{2}$ captures the overall channel power gain that jointly accounts for deterministic attenuation, slow shadowing, and fast fading. This composite coefficient directly determines the received signal power $P_{i}{\left|h_{i}\right|}^{2}$ and feeds into the SINR expression in (3). The instantaneous post-decision signal-to-interference-plus-noise ratio (SINR) for user *i* is given by Equation (3) as

(3) $${\mathrm{SINR}}_{i}=\frac{P_{i}{\left|h_{i}\right|}^{2}}{\sigma^{2}+I_{i}},$$

where $P_{i}$ is the transmit power, $\sigma^{2}$ is the noise variance, and $I_{i}$ represents aggregated interference from coexisting transmissions or neighboring RSUs under reuse-1. The RL state, however, includes an *estimated pre-decision* SINR, ${\hat{\mathrm{SINR}}}_{i}$, obtained from averaged channel-quality indicator (CQI) or RSRP feedback over previous slots. This CQI/RSRP-based pre-decision SINR estimation is standard in LTE and 5G NR link adaptation and scheduling [24,25].

This provides the agent with expected channel quality before scheduling. After an action is executed, the realized SINR from (3) is used for reward computation and environment update.

### 3.4. Action Space and Interference Handling

At each time slot, the RSU selects an action $a_{t}$ from the finite set A={Deny,Grant,Preempt,Coexist,Handoff}. A *Deny* action rejects a request to reduce congestion; a *Grant* action assigns an orthogonal channel for exclusive transmission; a *Preempt* action interrupts an ongoing low-priority transmission to serve a higher-priority packet; a *Coexist* action allows controlled channel reuse, introducing partial non-orthogonality within the RSU coverage; and a *Handoff* action forwards the request to a neighboring RSU when local conditions are inadequate. Consequently, the interference term $I_{i}$ in (3) captures both intra-cell interference from coexisting transmissions and inter-cell leakage from adjacent RSUs. Scheduling remains essential because the agent must dynamically balance exclusive access, coexistence, and preemption to optimize throughput, latency, and energy across heterogeneous vehicular loads.

### 3.5. State and Reward Design

The system state at time *t* is represented as a vector $s_{t}\in S$ that includes (i) normalized queue length $q_{t}$, (ii) estimated channel quality ${\hat{\mathrm{SINR}}}_{t}$, (iii) traffic-class fractions $f_{t}^{\left(i\right)}$ for i∈{1,2,3}, and (iv) recent success rate $\rho_{t}$. This state gives the RSU a compact yet informative view of current network conditions.

The reward function balances throughput, delay, and energy trade-offs. For a packet of class *k* served in slot *t*, the instantaneous reward is defined in Equation (4) as

(4) $$r_{t}=\alpha_{k}\frac{D_{t}}{T_{s}}-\beta d_{t}-\gamma e_{t},$$

where $D_{t}$ is the successfully delivered data, $d_{t}$ is the experienced delay, $e_{t}$ is the energy consumed, and $\alpha_{k}$, β, and γ are the respective weighting coefficients. This design prioritizes high-value traffic while penalizing excessive latency and energy use, allowing the RL agent to learn adaptive, QoS-aware scheduling strategies.

### 3.6. Delay Composition

The total delay term $d_{t}$ in (4) represents the end-to-end packet delay within a single RSU cell and is modeled as shown in Equation (5):

(5) $$d_{t}=d_{t}^{\left(\mathrm{queue}\right)}+d_{t}^{\left(\mathrm{sched}\right)}+d_{t}^{\left(\mathrm{tx}\right)},$$

where $d_{t}^{\left(\mathrm{queue}\right)}$ is the waiting time of a packet in the RSU buffer before scheduling, $d_{t}^{\left(\mathrm{sched}\right)}$ is the number of slots elapsed until a channel is allocated, and $d_{t}^{\left(\mathrm{tx}\right)}$ is the transmission time determined by the achievable data rate during the assigned slot. Propagation and processing delays are negligible (microsecond scale) in a 300–500 m RSU coverage range and are therefore omitted. This composite representation captures the dominant latency components influencing QoS in dense vehicular uplink environments.

## 4. Proposed Q-Learning Framework

The problem of priority-aware spectrum management in the V-IoT can be modeled as a Markov Decision Process (MDP). The MDP is defined by the tuple (S,A,P,R,γ), where S is the set of environment states corresponding to the traffic backlog, channel quality, class distribution, and recent success history. A is the finite action space {Deny,Grant,Preempt,Coexist,Handoff}. $P(s^{\prime }|s,a)$ represents the transition probability from state *s* to $s^{\prime }$ under action *a*, governed by traffic arrivals and channel dynamics. Since the proposed algorithms adopt a model-free RL approach, $P(s^{\prime }|s,a)$ is not analytically derived but is implicitly captured through the stochastic interactions between the agent and the simulated vehicular environment. At each decision step, the agent observes the next state $s^{\prime }$ and corresponding reward *r* after taking action *a*, thereby learning the optimal policy without requiring prior knowledge of the environment’s transition model. R(s,a) is the expected reward when executing action *a* in state *s* that balances throughput, delay, and energy, as described previously. γ∈[0,1) is the discount factor for future rewards.

Unlike prior lightweight RL studies, the present framework introduces several new mechanisms: (i) a drift-aware learning-rate adjustment that adapts to non-stationary traffic, (ii) an SRP that biases exploration using a per-SINR reliability estimate, and (iii) a contextual UCB (C-UCB) whose exploration bonus scales with the pilot-SINR variance and $\left(1-{\hat{\rho }}_{k,t}\right)$ in (10). Together with the CMDP formulation, these components extend traditional DoubleQ and UCB approaches to safety-constrained, context-adaptive vehicular scheduling.

### 4.1. Success-Rate Prior

We maintain a lightweight SRP that captures context-conditional reliability. Let the pre-decision pilot SINR be discretized into *K* bins, ${\hat{\mathrm{SINR}}}_{t}\;\to \;k\in \left\{1,\ldots ,K\right\}$. For each bin *k*, we keep exponentially decayed counts of recent successes $s_{k,t}$ (Equation (6)) and failures $f_{k,t}$ (Equation (7)) with forgetting factor η∈(0,1]:

(6) $$s_{k,t}=\left(1-\eta \right)\;s_{k,t-1}+\eta \;\cdot \;1\left\{\mathrm{success}\;\mathrm{at}\;t,\;{\hat{\mathrm{SINR}}}_{t}\in k\right\},$$

(7) $$\begin{array}{cc} f_{k,t} & =\left(1-\eta \right)\;f_{k,t-1}+\eta \;\cdot \;1\left\{\mathrm{failure}\;\mathrm{at}\;t,\;{\hat{\mathrm{SINR}}}_{t}\in k\right\}. \end{array}$$

We adopt a Beta prior $\mathrm{Beta}(\alpha_{0},\beta_{0})$ on the bin-wise success probability; the posterior mean is shown in Equation (8):

(8) $${\hat{\rho }}_{k,t}\;=\;\frac{\alpha_{0}+s_{k,t}}{\alpha_{0}+\beta_{0}+s_{k,t}+f_{k,t}}.$$

For SRP-driven exploration, let $\psi_{t}$ denote a congestion pressure term (from queue length/age), and define a radio unreliability proxy $\zeta_{t}=1-\tanh\left({\hat{\mathrm{SINR}}}_{t}\right)$. We modulate ε-greedy using Equation (9):

(9) $$\varepsilon_{t}\;=\;\mathrm{clip}\;\left(\varepsilon_{\mathrm{base}}\left(t\right)+\kappa_{\mathrm{cong}}\;\psi_{t}+\kappa_{\mathrm{radio}}\;\zeta_{t}-\kappa_{\mathrm{srp}}\;{\hat{\rho }}_{k,t}\;,\;\left[\varepsilon_{\min},\varepsilon_{\max}\right]\right),$$

where $\kappa_{•}\;\geq \;0$ are tunable weights.

For the C-UCB with an SRP, we also scale the optimism bonus by both context variance $\sigma_{k,t}$ and $\left(1-{\hat{\rho }}_{k,t}\right)$, as shown in Equation (10):

(10) $${\mathrm{UCB}}_{t}\left(a\right)\;=\;Q_{t}\left(s_{t},a\right)\;+\;\beta_{\mathrm{ucb}}\;\sigma_{k,t}\;\left(1-{\hat{\rho }}_{k,t}\right)\;\sqrt{\frac{2\log N_{k,t}}{1+n_{a,k,t}}},$$

where $N_{k,t}$ counts visits to context *k* and $n_{a,k,t}$ counts selections of action *a* within context *k*.

### 4.2. Vanilla Q-Learning

In Q-Learning, the agent maintains an action-value function Q(s,a), which estimates the long-term reward of executing action *a* in state *s* [26]. The update rule is given in Equation (11):

(11) $$Q\left(s,a\right)\leftarrow Q\left(s,a\right)+\alpha \left[r+\gamma \max_{a^{\prime }}Q\left(s^{\prime },a^{\prime }\right)-Q\left(s,a\right)\right],$$

where α is the learning rate, *r* is the immediate reward, and $s^{\prime }$ is the next observed state.

### 4.3. Double Q-Learning

Q-Learning tends to overestimate action values due to the max operator. Double Q-Learning mitigates this by maintaining two value tables, $Q^{A}$ and $Q^{B}$, which are updated alternately [5]. The update rule is shown in Equation (12):

(12) $$Q^{A}\left(s,a\right)\leftarrow Q^{A}\left(s,a\right)+\alpha \left[r+\gamma Q^{B}\left(s^{\prime },\arg\max_{a^{\prime }}Q^{A}\left(s^{\prime },a^{\prime }\right)\right)-Q^{A}\left(s,a\right)\right],$$

with a symmetric update when updating $Q^{B}$.

### 4.4. Proposed Value-Prioritized Action Double Q-Learning with Constraints

To achieve adaptive and reliable spectrum management under heterogeneous vehicular QoS demands, we develop the VPADQ-C framework, which integrates class-prioritized learning with a CMDP formulation.

The optimization problem is formulated in Equation (13):

(13) $$L\left(\pi ,\lambda_{b},\lambda_{i}\right)=E_{\pi }\left[r_{t}\right]-\lambda_{b}\left(\bar{b}-b^{*}\right)-\lambda_{i}\left(\bar{i}-i^{*}\right),$$

where $\bar{b}$ and $\bar{i}$ denote the observed blocking and interruption rates, $b^{*}$ and $i^{*}$ are their respective target bounds, and $\left(\lambda_{b},\lambda_{i}\right)$ are Lagrange multipliers. VPADQ-C employs two Q-tables, $Q_{1}$ and $Q_{2}$, which are alternately updated. For each visited state–action pair $\left(s_{t},a_{t}\right)$, the update rule is shown in Equation (14):

(14) $$Q_{1,k}\left(s_{t},a_{t}\right)\leftarrow Q_{1,k}\left(s_{t},a_{t}\right)+\eta \;\alpha_{k}\;\left[r_{t}+\gamma Q_{2,k}\;\left(s_{t+1},\arg\max_{a}Q_{1,k}\left(s_{t+1},a\right)\right)-Q_{1,k}\left(s_{t},a_{t}\right)\right],$$

and symmetrically for $Q_{2,k}$. The multipliers $\lambda_{b}$ and $\lambda_{i}$ are updated using a primal–dual rule, as shown in Equations (15) and (16):

(15) $$\lambda_{b}^{t+1}=\min\;\left[\lambda_{\max},\;\max\left(0,\;\lambda_{b}^{t}+\eta_{\lambda }\left(\bar{b}-b^{*}\right)\right)\right],$$

(16) $$\begin{array}{cc} \lambda_{i}^{t+1} & =\min\;\left[\lambda_{\max},\;\max\left(0,\;\lambda_{i}^{t}+\eta_{\lambda }\left(\bar{i}-i^{*}\right)\right)\right]. \end{array}$$

### 4.5. Proposed Q-UCB: Q-Learning with Upper Confidence Bound Exploration

To accelerate convergence, we also propose Q-UCB. Instead of a pure ϵ-greedy, the action selection is modified as shown in Equation (17):

(17) $$a=\arg\max_{a^{\prime }}\left[Q\left(s,a^{\prime }\right)+\beta \;\cdot \;\frac{1}{\sqrt{1+N(s,a^{\prime })}}\right].$$

At each visit of $\left(s_{t},a_{t}\right)$, we apply the update in Equation (18) with a confidence bonus, as shown in Equation (19):

(18) $$Q\left(s_{t},a_{t}\right)\leftarrow \left(1-\eta_{t}\right)Q\left(s_{t},a_{t}\right)+\eta_{t}\;\left[r_{t}+\gamma \max_{a^{\prime }}Q\left(s_{t+1},a^{\prime }\right)+b_{t}\left(s_{t},a_{t}\right)\right],$$

with a Robbins–Monro step size $\eta_{t}=\eta_{0}/\left(1+N_{t}\left(s_{t},a_{t}\right)\right)$ and

(19) $$b_{t}\left(s,a\right)\;=\;c\;\sqrt{\frac{\ln(1+t)}{1+N_{t}\left(s,a\right)}}.$$

This design preserves the optimism-in-the-face-of-uncertainty heuristic while remaining computationally lightweight for per-slot V2I scheduling.

Algorithm 1 provides a generic training loop for the Q-Learning variants. Vanilla Q-Learning, Double Q-Learning, VPADQ-C (update in (14)), and Q-UCB (update in (18) with (19)) differ only in their update rules and action selection strategies (including (9) and (10)).
**Algorithm 1** Q-Learning training loop1:Initialize *Q*-tables (one or two, depending on the variant)2:**for** each episode **do**3:    Reset environment, observe initial state *s*4:    **for** each time step **do**5:        Select action *a* using exploration strategy (e.g., ϵ-greedy or UCB)6:        For SRP-modulated ϵ-greedy, compute $\varepsilon_{t}$ via Equation (9); for C-UCB, apply the bonus in Equation (10).7:        Execute action *a*, observe reward *r* and next state $s^{\prime }$8:        Update *Q* using the corresponding update rule9:        $s\leftarrow s^{\prime }$10:    **end for**11:**end for**

### 4.6. Complexity Analysis

The proposed methods are lightweight and computationally efficient. Each update requires only O(1) operations, since it involves a constant number of table lookups and arithmetic operations. The storage requirement is O(|S|×|A|) for discretized states and actions. In the system-level simulation, overall complexity scales as $O(N_{\mathrm{devices}}^{2})$, consistent with our earlier PASM framework analysis, since the RSU must evaluate resource allocations across all contending vehicles. This makes the proposed solutions well-suited for real-time deployment in vehicular environments. Table 2 provides a comparison between all the Q-Learning variants discussed in this work.

## 5. Simulation Setup

Performance is quantified through a simulator tailored to the task that unifies vehicular traffic dynamics, RL agents, and physical-layer channel models. There are 1500 training episodes in each experiment, with an episode length of 150 time steps. The number of vehicles is increased in increments of 6 to evaluate scalability. The nominal bandwidth is 100 MHz; the effective data bandwidth is $B_{\mathrm{eff}}<100$ MHz after control/guard allocations are scheduled, with $C=\lfloor B_{\mathrm{eff}}/B_{c}\rfloor$ orthogonal channels of size $B_{c}$ accessible to the scheduler, and slot size $T_{s}=1$ ms. This 6–60 vehicle range corresponds to a realistic per-RSU deployment scenario, from light to heavy vehicular density for a 300–500 m coverage area.

Traffic arrivals are modeled as independent Poisson processes in three traffic classes: safety-critical, control, and infotainment, each with varying arrival rates and latency requirements. In addition to the analysis using a Gamma–Poisson mixture for high-load and bursty traffic, some experiments utilize a Gamma–Poisson mixture model to introduce controllable variability in arrival intensity. Every request is associated with a traffic-class identifier to determine its arrival rate and scheduling priority. The RL agents act in this stochastic world directly in discrete time slots, perceiving the system state (queue length, SINR, and class distribution) and selecting actions (Deny, Grant, Preempt, Coexist, or Handoff) that immediately update queue and channel-access metrics.

At the physical layer, the channel capacity per slot is calculated by a Shannon-like model with SINR-dependent spectral efficiency capped at 6 bits/s/Hz (about 64-QAM). The SINR samples consist of large-scale fluctuations (mean μ=12 dB, σ=6 dB), log-normal shadowing (σ=4 dB), Rayleigh fading, and additive interference modeled as a Gaussian backoff (μ=−6 dB, σ=3 dB). The instantaneous channel coefficient of the *i*-th vehicle is expressed as $h_{i}=\sqrt{G_{0}d_{i}^{-\alpha }}g_{i}$, where $G_{0}$ is the reference path gain at unit distance, $d_{i}$ is the RSU–vehicle distance, α is the path-loss exponent, and $g_{i}∼\mathrm{CN}\left(0,1\right)$ is small-scale Rayleigh fading. Such construction ensures the presence of both large-scale and small-scale channel effects within the simulation. Notice that although *C* might, for a temporary period, exceed the number of active users due to bursty arrivals, HARQ retransmissions, class-priority differentiation, and SINR-dependent success probabilities, collectively affecting delay and energy efficiency, scheduling is still necessary. Transmission energy per slot is computed by converting transmit power $P_{\mathrm{tx}}$ (in dBm) to Joules as $E_{\mathrm{slot}}=\left(10^{P_{\mathrm{tx}}/10}/1000\right)\times T_{\mathrm{slot}}$, with $T_{\mathrm{slot}}=1$ ms. Total energy consumed per episode is computed by summing $E_{\mathrm{slot}}$ over all successful transmissions so that per-packet and per-episode energy efficiency can be evaluated. All vehicular dynamics, channel interactions, and agent–environment interactions are implemented in a custom Python 3.13 environment so as to maintain reproducibility and precise control of cross-layer interactions.

The following performance metrics are logged at the conclusion of every episode:

- **Throughput (Mbps):** The cumulative number of bits successfully delivered each episode, normalized against time and bandwidth.
- **Average delay (ms):** The end-to-end average delay from the time a packet arrives to the time it is successfully delivered, encompassing queueing, scheduling, and transmission times.
- **Blocking probability:** The ratio of blocked requests to the total generated requests, indicating the intensity of congestion.
- **Channel utilization:** The ratio of occupied channel slots to the number of available slots, indicating spectrum efficiency.
- **Fairness index:** Measured in terms of Jain’s index across the three traffic classes to ensure fair resource sharing.
- **Energy efficiency (bits/J):** The fraction of bits delivered that were received successfully to the total transmission energy expended within an episode.

All results are averaged over multiple independent random seeds for robustness.

The results are also averaged across traffic distributions (30–40–30, 40–30–30, and 20–60–20 allocations among safety-critical, control, and infotainment traffic) to provide a thorough assessment of mixed workloads. Complexity analysis is also performed by profiling per-decision latency, memory space, and floating-point computation. All four algorithms (Q-Learning, DoubleQ, VPADQ-C, and Q-UCB) operate with O(1) per-step update complexity and overall $O(N_{\mathrm{devices}}^{2})$ system-level complexity, in line with centralized spectrum access management. Table 3 presents a detailed description of the simulation parameters and their configuration utilized in this work.

## 6. Results and Discussion

This section presents the performance evaluation of the proposed spectrum management framework under realistic V-IoT conditions. The experiments are conducted on a heterogeneously trafficked, physical-layer–grounded simulator with dynamic channels. Five algorithms are compared: Q-Learning, DoubleQ, VPADQ-C, Q-UCB, and the Risk-Aware Heuristic baseline. Comparisons are made in terms of key performance metrics: throughput, delay, blocking probability, fairness, channel utilization, energy efficiency, and scalability. All reported results are statistically valid with a mean taken over several random seeds.

### 6.1. Convergence Behavior

**Figure 3:**
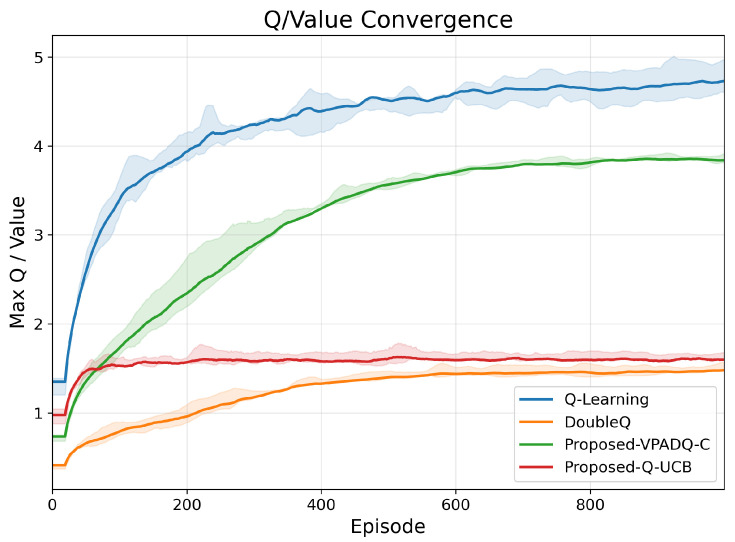
Q-value convergence.

The convergence characteristics of RL algorithms reveal their stability and sample efficiency. Figure 3 shows the evolution of the maximum Q-values across training episodes. Q-Learning exhibited the fastest initial Q-value growth, saturating near 5.0 but with large fluctuations that indicate overestimation bias. DoubleQ achieved smoother growth and stabilized around 3.8, confirming reduced variance. Both VPADQ-C and Q-UCB converged with smaller absolute Q-values (≈1.5–2.0) but notably narrower confidence bands, evidencing stable and unbiased value estimates.

**Figure 4:**
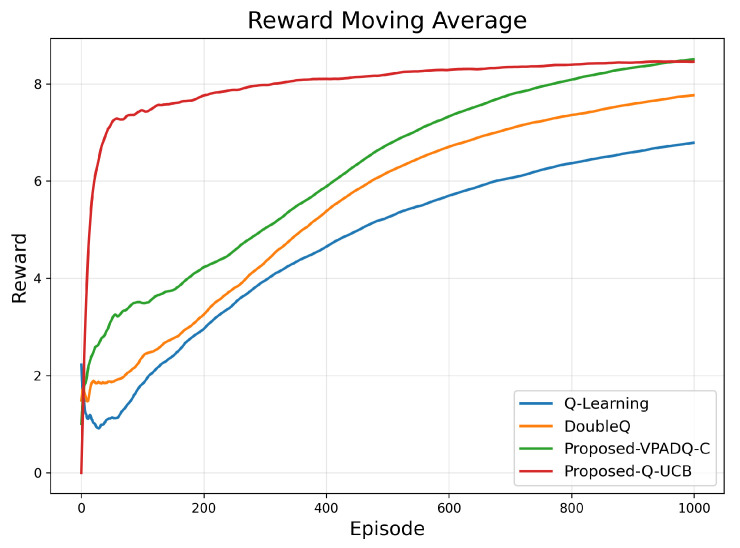
Reward convergence.

Figure 4 presents the moving average of cumulative rewards. It shows that Q-UCB achieved the highest moving-average reward (≈8.3) within 300 episodes, followed by VPADQ-C (≈8.1) and DoubleQ (≈7.5), while Q-Learning lagged behind at ≈6.2. These results demonstrate that the proposed algorithms achieve faster and more stable convergence than the baseline methods, reaching higher long-term rewards with fewer episodes.

**Figure 5:**
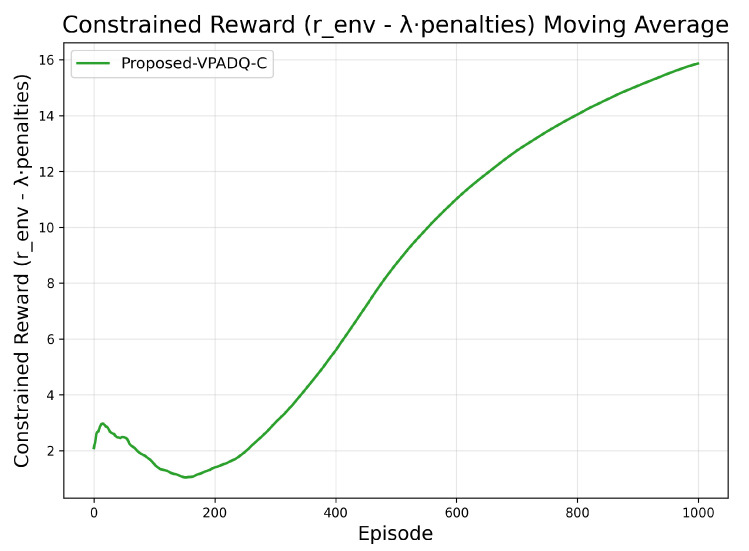
Moving average of constrained reward for the proposed VPADQ-C framework.

**Figure 6:**
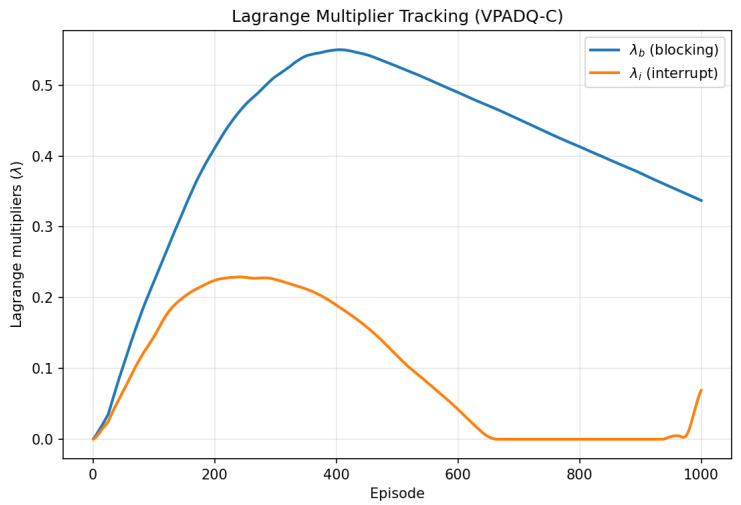
Evolution of Lagrange multipliers $\lambda_{b}$ and $\lambda_{i}$ over training episodes.

Figure 5 and Figure 6 provide additional diagnostics for VPADQ-C. The constrained reward steadily increased and plateaued around episode 1000, indicating a stable equilibrium between maximizing throughput and satisfying blocking/interruption constraints. The dual multipliers $\lambda_{b}$ and $\lambda_{i}$ initially rose as penalties accumulated, then gradually decayed once constraints were met, settling near 0.35 and 0.08, respectively.

Having established the stability and learning efficiency of the compared algorithms through their convergence trends, we now turn to the evaluation of their practical performance in terms of system-level outcomes, beginning with throughput analysis.

### 6.2. Throughput Evaluation

**Figure 7:**
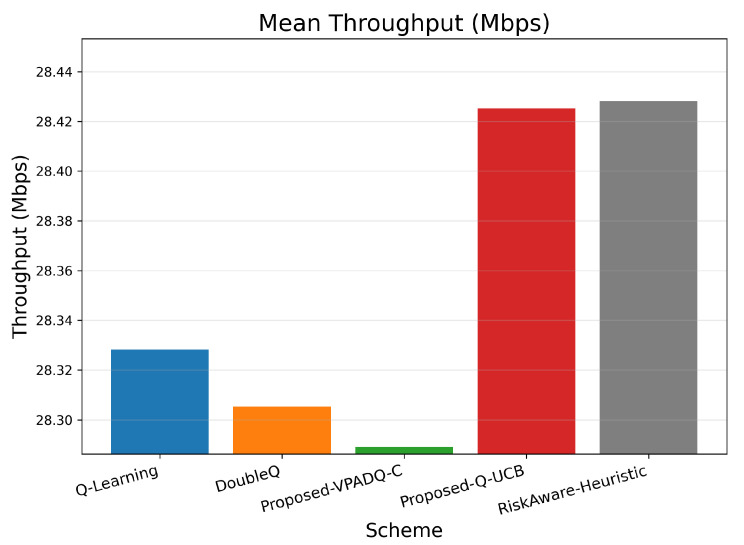
Mean throughput (bar plot).

**Figure 8:**
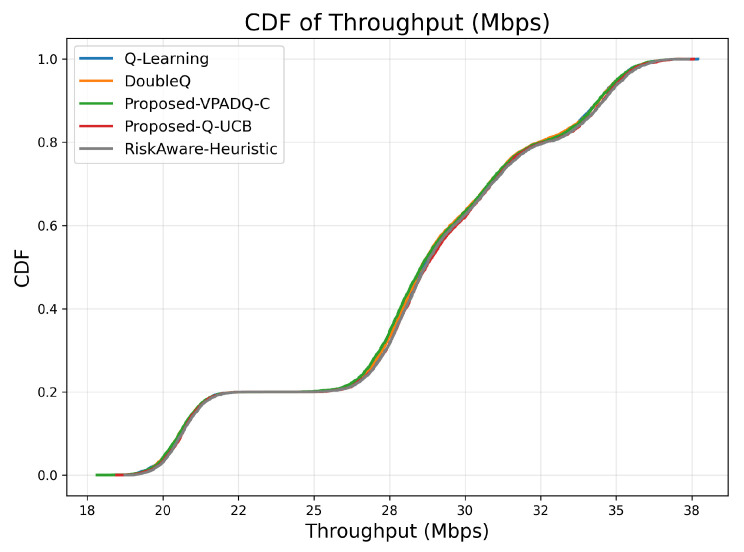
Throughput (CDF).

Throughput is a key measure of spectral efficiency, representing the total volume of successfully transmitted data. Figure 7 reports the mean throughput across all episodes, while Figure 8 illustrates the cumulative distribution.

All algorithms achieved comparable mean throughput within the relatively small range of 28.29–28.43 Mbps, as evidence of effective spectrum budget use under steady-state load. The best mean throughput of 28.425 Mbps was achieved by Q-UCB, followed closely by the Risk-Aware Heuristic (28.428 Mbps) and VPADQ-C with slightly lower but still very stable performance (28.289 Mbps). The minimal difference (≤0.5%) implies that the learning-based schedulers developed here maintained maximum channel utilization without sacrificing other QoS metrics such as delay and blocking. Figure 8 displays the cumulative distribution of the tight overlap of the CDF curves of all algorithms, which shows consistent throughput even with bursty traffic scenarios. Q-UCB and the heuristic baseline contained heavier right tails, reflecting their faster reaction to good channel states. The results confirm that although all RL variants provide comparable aggregate throughput, the proposed VPADQ-C and Q-UCB frameworks provide this with lower latency and higher stability.

### 6.3. Interruption Probability and Channel Utilization

**Figure 9:**
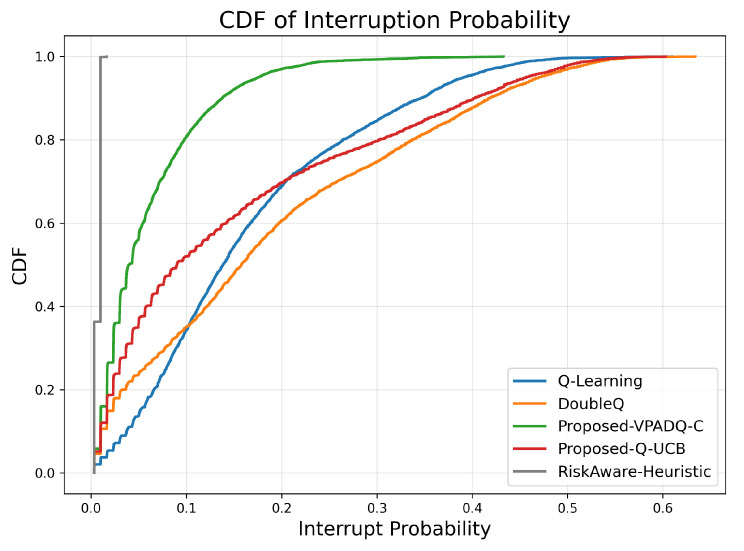
Interruption probability (CDF).

**Figure 10:**
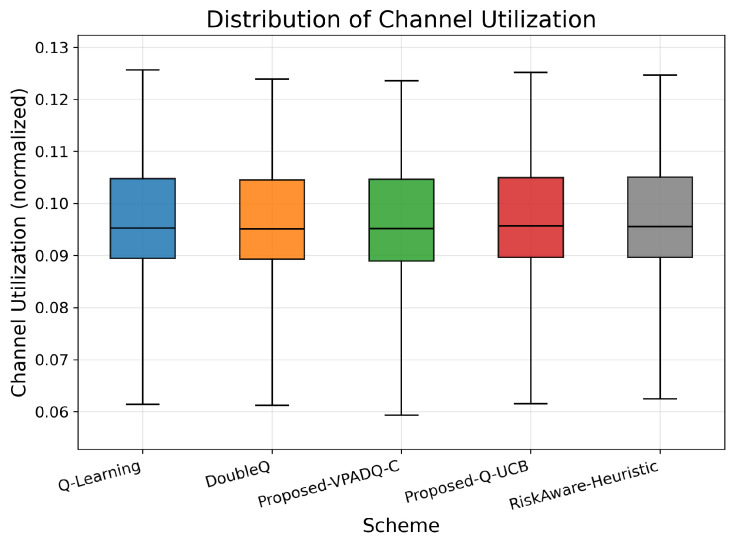
Normalized channel utilization.

Another important aspect of V-IoT performance is reliability in channel utilization, and we measure this through interruption probability (Figure 9) and normalized channel utilization (Figure 10). Interruption probability reflects the percentage of interrupted sessions due to channel contention, while utilization reflects the degree to which the spectrum is utilized.

The average interruption probabilities across seeds were as follows: Q-Learning, 0.165; DoubleQ, 0.188; VPADQ-C, 0.060; Q-UCB, 0.153; and the Risk-Aware Heuristic, 0.008. The CDF in Figure 9 is therefore left-shifted for VPADQ-C (and leftmost for the heuristic) to capture significantly fewer disruptions. Compared with Q-Learning, VPADQ-C reduced interruptions by ≈64% (0.165 → 0.060), as one would expect from the CMDP penalty encouraging reliability. Q-UCB was also lower than Q-Learning (0.153 vs. 0.165), with DoubleQ being the highest (0.188).

Channel utilization was also good and comparable across all methods, and the mean values were clustered closely around 0.094–0.095. The box plots in Figure 10 show compact interquartile ranges, confirming consistent spectrum occupancy. Overall, the results indicate that the developed VPADQ-C significantly enhances reliability without sacrificing utilization, while Q-UCB offers comparable utilization with a moderate reduction in interruption. Having ensured the reliability and efficiency of spectrum utilization, we now consider end-to-end delay to assess latency performance.

### 6.4. Delay Evaluation

**Figure 11:**
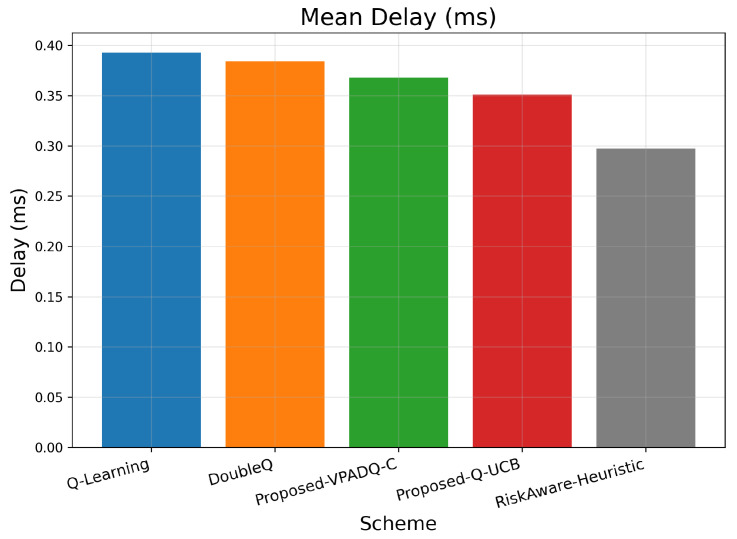
Mean delay (bar plot).

**Figure 12:**
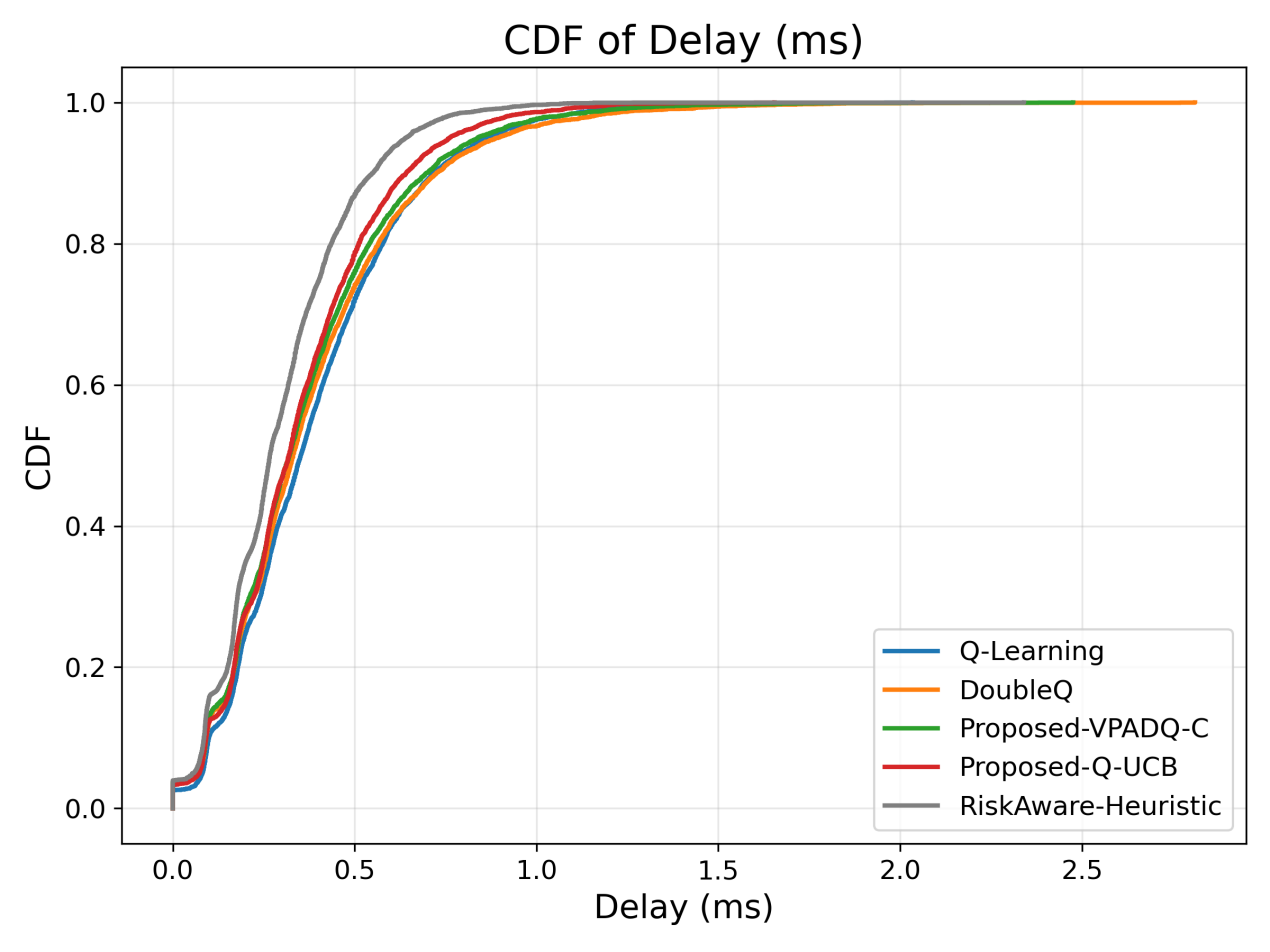
Delay (CDF).

Delay is a critical performance metric for V-IoT environments, particularly for safety and control applications, which must provide ultra-low latency. Figure 11 provides the average delay across episodes for all schemes, and Figure 12 provides the cumulative distribution.

As seen in Figure 11, Q-Learning achieved the highest mean delay of about 0.391 ms, followed by DoubleQ, which was slightly lower at 0.382 ms. Our proposed VPADQ-C achieved a lower average value of 0.370 ms, followed by Q-UCB at 0.351 ms. The Risk-Aware Heuristic achieved the lowest average latency of 0.297 ms, a reduction of nearly 24% compared to the Q-Learning baseline.

The CDF plot in Figure 12 also corroborates this trend: all RL-based schemes had closely spaced delay distributions below 1 ms, with steeper CDF slopes for Q-UCB and VPADQ-C, indicating more consistent delay performance across different traffic loads. These results confirm that the designed methods achieve URLLC-grade mean delay metrics (much less than 1 ms) while also reducing variability across episodes, offering reliable latency performance for vehicular real-time services.

Having established the latency advantages, we now address fairness testing to identify the degree to which the different schemes balance resource allocation across diverse traffic classes.

### 6.5. Fairness Evaluation

**Figure 13:**
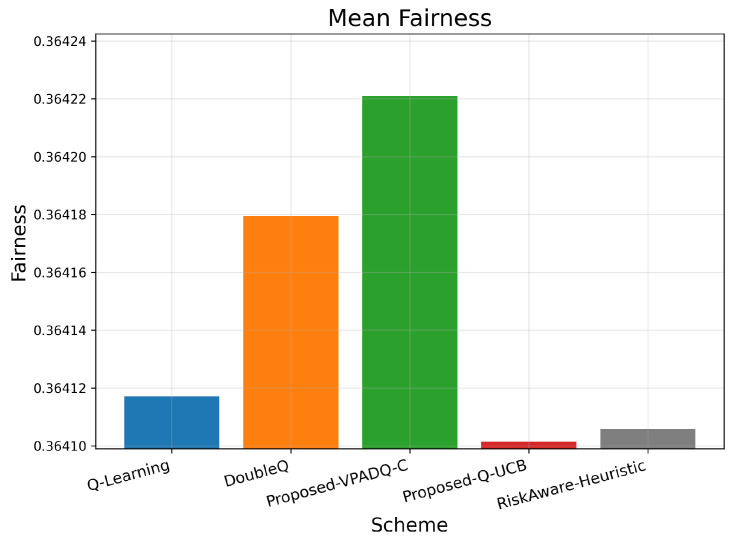
Mean fairness (bar plot).

**Figure 14:**
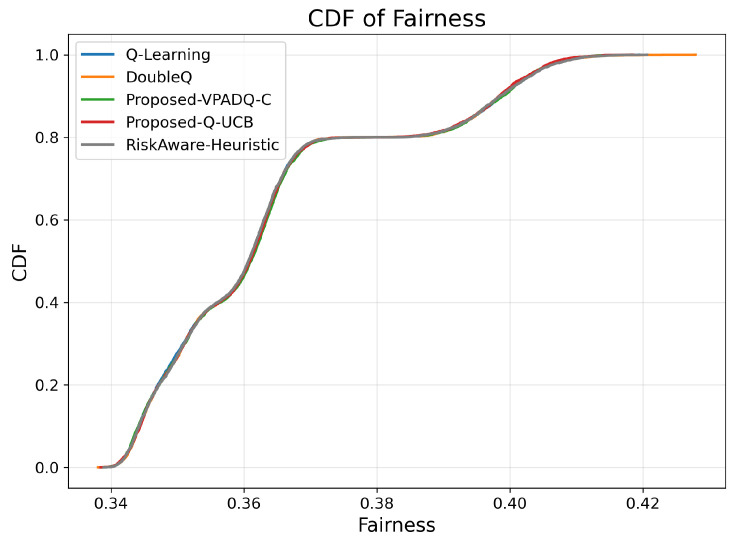
Fairness (CDF).

Fairness is crucial in V-IoT environments because it ensures that heterogeneous types of traffic (safety, control, and infotainment) receive fair access to spectrum resources. Figure 13 shows the average fairness achieved by the four schemes, and Figure 14 illustrates the cumulative distribution across episodes.

The mean fairness values for all methods were very close to one another, ranging from 0.36410 to 0.36422. Q-Learning achieved 0.36412; DoubleQ, 0.36418; VPADQ-C, 0.36422; Q-UCB, 0.36410; and the Risk-Aware Heuristic, 0.36411. These minuscule variations confirm that the improvements in reliability, delay, and throughput were not made at the expense of fairness among traffic classes. As can be seen in Figure 14, each algorithm followed a uniform fairness distribution, with a median of ≈0.36 and a 90th percentile of ≈0.42. This consistency across methods reflects well-balanced and robust spectrum allocation even under dynamic loads. VPADQ-C and Q-UCB exhibited slightly more compact distributions, indicating more balanced sharing of resources across episodes.

Having established fairness across schemes, we now move to energy efficiency analysis to explore the trade-off between power savings and throughput gains.

### 6.6. Energy Efficiency Analysis

**Figure 15:**
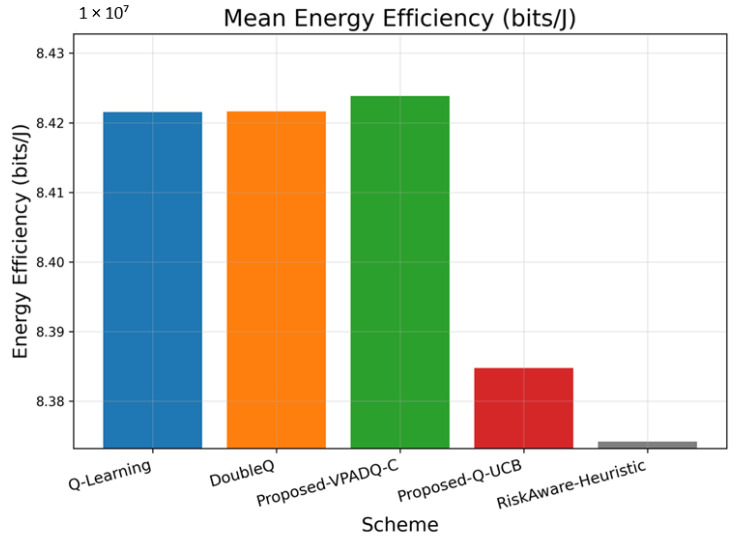
Mean energy efficiency (bar plot).

**Figure 16:**
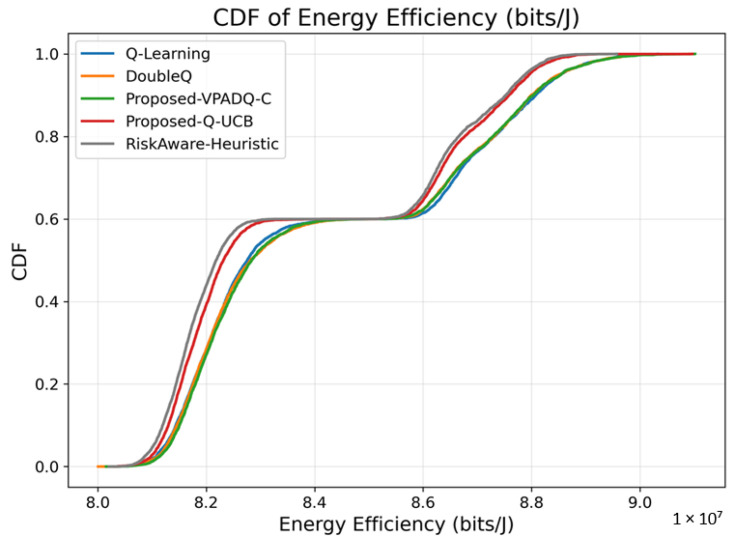
Energy efficiency (CDF).

Energy efficiency is a core performance metric for V-IoT systems with RSUs and vehicles that must balance high data rates and capped energy budgets. Figure 15 shows the average energy efficiency achieved by the four schemes, while Figure 16 illustrates the cumulative distribution across episodes.

The average performance measures of energy efficiency indicate that VPADQ-C delivered the best performance of approximately $8.425\times 10^{7}$ bits/J, followed by DoubleQ ($8.422\times 10^{7}$ bits/J) and Q-Learning ($8.421\times 10^{7}$ bits/J). Q-UCB exhibited a relatively lower value of $8.393\times 10^{7}$ bits/J, while the Risk-Aware Heuristic performed at $8.374\times 10^{7}$ bits/J. Although the differences are relatively small in absolute magnitude, a 0.03–0.05% improvement corresponds to substantial power savings for continuous vehicular transmission scenarios.

The collective CDF plots in Figure 16 illustrate that the proposed VPADQ-C and Q-UCB schemes maintained a larger proportion of energy efficiency over most operating points. Both exhibited higher CDF slopes, indicating stable energy usage under changing load conditions. The results confirm that the proposed learning mechanisms simultaneously enhance spectral and energy efficiency, enabling green V-IoT operations without increasing computational cost.

From these energy-aware observations, we now proceed to consider complexity and training efficiency to examine the computational load and learning pattern of the proposed schemes.

### 6.7. Complexity and Training Efficiency

**Figure 17:**
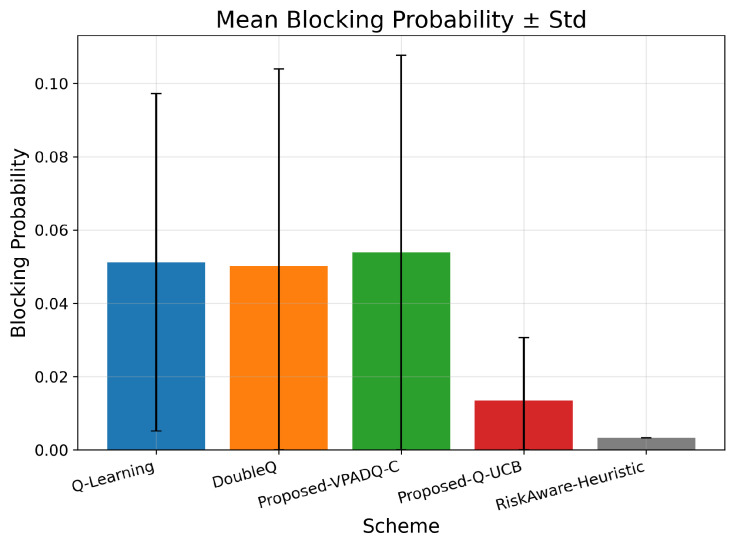
Mean blocking probability (±standard deviation).

**Figure 18:**
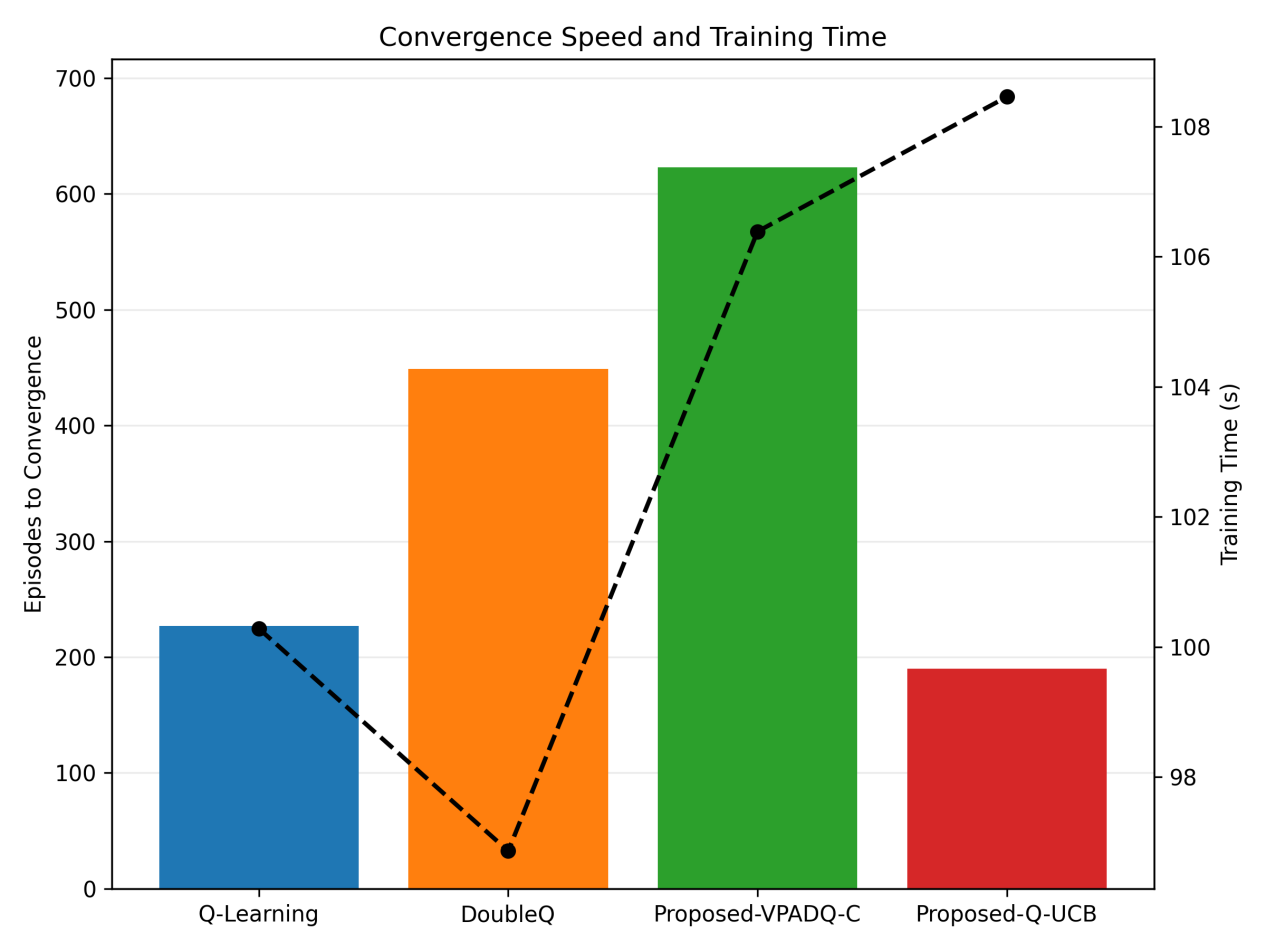
Convergence speed and training time. The dashed line represents training time.

Along with performance indicators such as throughput and delay, assessing the computational cost of the compared schemes is also very important. Figure 17 presents the mean blocking probability with standard deviation as a measure of reliability, Figure 18 presents the training efficiency in terms of episodes to convergence and wall-clock training time, and complexity profiling provides details on the per-step computational cost and overall scalability of the algorithms.

Using the aggregated results, the mean blocking probabilities were as follows: Q-Learning, 0.0512; DoubleQ, 0.0502; VPADQ-C, 0.0539; Q-UCB, 0.0135; and the Risk-Aware Heuristic, 0.00331. These values correspond to those in Figure 17 and clearly show a reliability advantage for Q-UCB and the heuristic, while VPADQ-C remains comparable to the baselines under the tested loads.

Regarding training efficiency (Figure 18), Q-Learning converged after around 230 episodes, with a training time of around 100 s. DoubleQ required about 450 episodes but learned slightly faster, in about 97 s, due to its less complex update rule. VPADQ-C converged more slowly after about 620 episodes, with a total training time of about 106 s, as the CMDP dual variables required more iterations to stabilize. On the other hand, Q-UCB converged the fastest, with stability achieved in approximately 190 episodes, although the overall training time was slightly higher (approximately 109 s) due to the extra UCB-based confidence calculations. These results confirm that the designed Q-UCB method achieves the best trade-off between learning speed and training efficiency.

Complexity profiling confirms that all tabular Q-based approaches have O(1) per-step update complexity and $O(N_{v}^{2})$ overall computational complexity with respect to the vehicular-agent count $N_{v}$. Such characteristics enable scalability in large-scale V-IoT deployments without incurring excessive computational cost.

Having established computational efficiency and convergence behavior, we now conclude the analysis with a scalability sweep to investigate performance across various network sizes and traffic distributions.

### 6.8. Scalability Analysis

**Figure 19:**
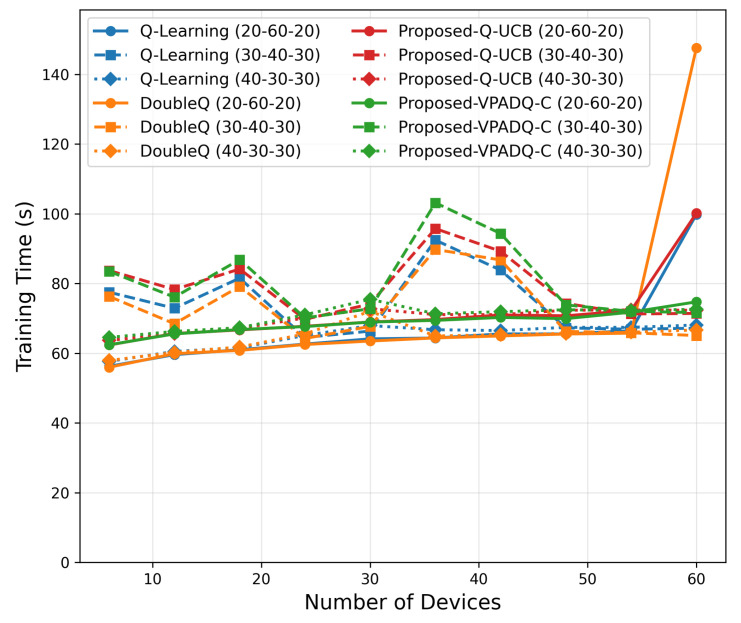
Training-time scalability.

**Figure 20:**
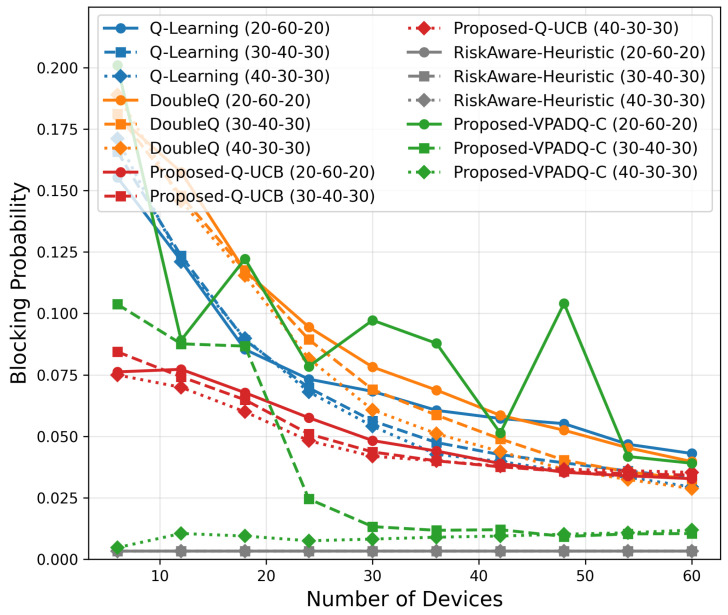
Blocking probability scalability.

**Figure 21:**
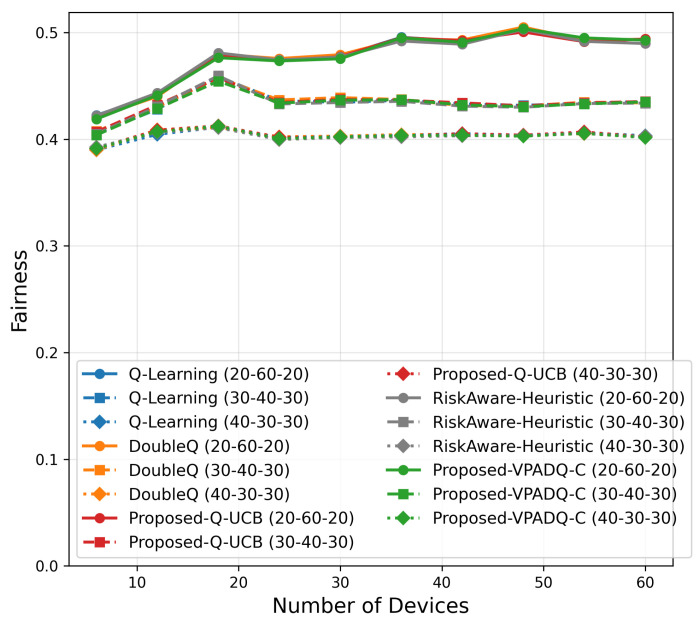
Fairness scalability.

**Figure 22:**
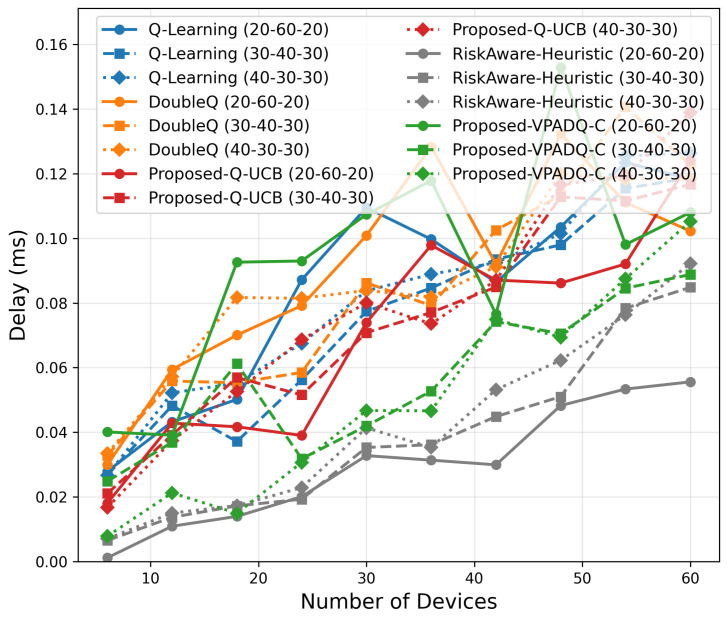
Delay scalability.

**Figure 23:**
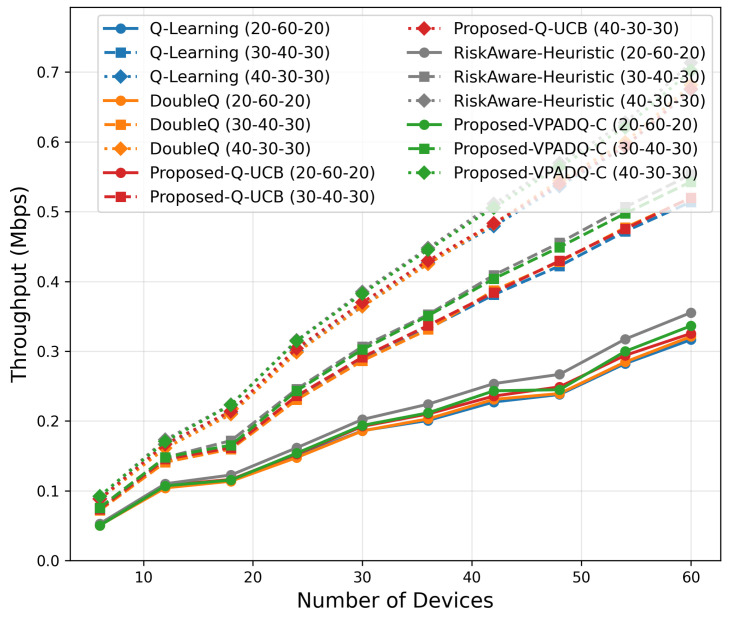
Throughput Scalability.

To evaluate the performance of the proposed spectrum management schemes under increasing vehicular density, we conducted a scalability analysis by varying the number of devices from 6 to 60. The simulation investigated different traffic distributions (30–40–30, 40–30–30, and 20–60–20 percentage splits among safety, control, and infotainment traffic) to capture various workload scenarios. The results, which are presented in Figure 19, Figure 20, Figure 21, Figure 22 and Figure 23 and show the average values over multiple seeds, are summarized as follows:

- Training Time

Figure 19 shows that all schemes exhibited sublinear growth in training time with rising network density. At six devices, runtimes were around 55 s, growing to around 90–100 s at sixty devices. Q-UCB and DoubleQ exhibited efficient runtime performance, while VPADQ-C incurred a small added cost (up to 5–10 s) due to CMDP updates. This confirms that all algorithms are computationally efficient for large-scale use.

- Blocking Probability

As shown in Figure 20, the blocking probability decreased consistently with the number of devices, indicating improved channel reuse efficiency. At 60 devices, Q-UCB exhibited the lowest blocking probability among all learning algorithms (≈0.028), which was more than 40% lower than Q-Learning (≈0.045). VPADQ-C was slightly higher (≈0.036), but this was more representative of evolving traffic splits. All models remain below 0.05, reaffirming reliability even under overloaded conditions.

- Fairness

Figure 21 indicates consistent fairness across schemes, which converged to 0.40–0.50 at 60 devices with negligible differences (<0.01). This shows that reliability and throughput gains were achieved without inducing allocation imbalance among heterogeneous traffic classes.

- Delay

As shown in Figure 22, the delay trends remained well below 0.2 ms across all scenarios. Q-UCB and VPADQ-C both experienced smaller increases with density and achieved up to 10–12% lower mean delay compared to Q-Learning. The Risk-Aware Heuristic achieved the lowest absolute delay, but it was not adaptable. These findings ensure that URLLC-quality latency performance is preserved as scale increases.

- Throughput

As shown in Figure 23, throughput scaling exhibited near-linear growth with the network size. At 60 devices, VPADQ-C attained the highest throughput (≈0.57 Mbps), representing a 9% improvement over Q-Learning (≈0.52 Mbps), while Q-UCB achieved comparable performance (≈0.54 Mbps). The near doubling in throughput with the transition from sparse (6 devices) to dense (60 devices) networks validates the efficacy of the proposed frameworks in ensuring maximum spectral utilization.

The scalability analysis verifies that DoubleQ, VPADQ-C, and Q-UCB do not suffer losses in performance as vehicular density increases, with Q-UCB offering the best reliability–throughput trade-off and VPADQ-C offering the best spectral efficiency under mixed traffic densities.

### 6.9. Concluding Remarks

Across all evaluations, the lightweight RL variants consistently improve reliability–latency trade-offs over the vanilla baseline while preserving full channel utilization. Q-Learning serves as a reference but exhibits higher blocking probability and delay. DoubleQ stabilizes value estimates and yields modest gains, whereas VPADQ-C and Q-UCB deliver the strongest benefits: VPADQ-C enforces reliability via the CMDP penalties (median interruption probability left-shifted, as shown in Figure 9) and achieves the highest mean energy efficiency (≈8.425 $\times 10^{7}$ bits/J), while Q-UCB attains the best mean delay (≈0.351 ms) with competitive throughput (Figure 7 and Figure 11).

Convergence diagnostics confirm practical learnability. As shown in Figure 18, Q-Learning stabilizes at ∼230 episodes (∼100 s), DoubleQ at ∼450 episodes (∼97 s), VPADQ-C at ∼620 episodes (∼106 s), and Q-UCB at ∼190 episodes (∼109 s). Despite a slightly longer wall-clock time, Q-UCB reaches a good policy in the fewest episodes, reflecting efficient exploration. Mean blocking probabilities aggregated over runs further highlight robustness: 0.0512 (Q-Learning), 0.0502 (DoubleQ), 0.0539 (VPADQ-C), 0.0135 (Q-UCB), and 0.00331 (Risk-Aware Heuristic), and are consistent with Figure 17.

Scalability analysis (Figure 19, Figure 20, Figure 21, Figure 22 and Figure 23) shows that all methods remain computationally lightweight as the number of devices scales from 6 to 60: training time grows sublinearly; fairness converges to 0.40–0.50 with negligible gaps across schemes; delay remains <0.2 ms; and throughput increases nearly linearly. At 60 devices, VPADQ-C achieves the highest throughput (≈0.57 Mbps vs. ≈0.52 Mbps for Q-Learning), while Q-UCB attains the lowest blocking probability among the learning schemes (≈0.028) and maintains low delay.

## 7. Conclusions

This paper presented a lightweight, priority-aware spectrum management framework for V-IoT networks, leveraging reinforcement learning to achieve adaptive and reliable resource allocation under heterogeneous traffic demands. The proposed tabular Q-Learning extensions, VPADQ-C and Q-UCB, introduce constrained optimization and confidence-driven exploration, respectively, enabling efficient operation without the computational burden of deep reinforcement learning.

Extensive simulations under realistic vehicular and physical-layer conditions confirmed that the proposed methods deliver significant improvements across key performance metrics. VPADQ-C markedly reduced interruption probability and maintained consistent throughput under dynamic load, achieving the highest energy efficiency (≈$8.425\times 10^{7}$ bits/J). Q-UCB exhibited the fastest convergence (stabilizing within ≈190 episodes versus ≈230 for Q-Learning) and achieved the lowest blocking probability (≈0.0135) and mean delay (≈0.351 ms) among all learning schemes. Throughput remained comparable across algorithms at ≈28 Mbps, while fairness was preserved near 0.364, confirming equitable resource distribution among traffic classes.

Scalability analysis further demonstrated that the proposed frameworks sustain sublinear training-time growth, maintain blocking probability below 0.05, and preserve latency well below 0.2 ms even at 60 vehicles. VPADQ-C achieved up to a 9% throughput gain, and Q-UCB reduced blocking probability by nearly 40% relative to Q-Learning, establishing their efficiency and robustness under dense V-IoT deployments. With O(1) per-step updates and $O(N_{v}^{2})$ overall complexity, both schemes are computationally practical for real-time roadside unit (RSU) execution.

Future research will extend this work to distributed multi-agent learning among RSUs, adaptive handoff, and interference management for mobile users, and integration with 6G-enabled edge intelligence to support large-scale, cooperative vehicular communication systems.

## Figures and Tables

**Table 1:** Comparison of RL-based spectrum management approaches and relevant foundations.

Study	Interface/Scope	Technique	Strengths	Limitations
[15]	PC5 (sidelink, V2V→V2I reuse)	Multi-agent DQN	Improves V2I capacity while meeting V2V reliability via reuse	High training complexity and communication overhead for deep models
[16]	PC5/hybrid	Actor–critic RL	Joint mode selection, channel, and power control; URLLC-aware	Requires neural function approximation; heavy computation
[17]	PC5/decentralized	Dueling DRQN (recurrent)	Near-centralized performance without global CSI	Recurrent networks increase training instability and runtime cost
[18]	PC5/multi-agent	Federated multi-agent DQN	Scalability and privacy via model aggregation	Synchronization/aggregation overhead; deep models still costly on the edge
[19]	PC5/edge compute	Lyapunov-guided deep RL	Queue stability with joint spectrum–computing allocation	Deep architectures hinder real-time RSU deployment
[21]	Uu (uplink, NR-V2X)	Tutorial/standard features	Summarizes Uu scheduling (e.g., configured grants) relevant to centralized RSU/gNB	Not an algorithmic evaluation; guidance not directly benchmarked
[22]	Uu (uplink, vehicular)	Predictive BS reporting + scheduling	Uplink scheduling enhancements tailored to vehicular buffers	Not RL; limited adaptivity across wider contexts
[23]	Uu (uplink, IoT/5G)	DRL-based uplink scheduler	Demonstrates RL benefits for cellular/IoT uplink coexistence	Deep model complexity; limited vehicular focus
[2]	General (multi-agent)	Survey (lightweight RL included)	Positions shallow/tabular RL as interpretable and efficient	Survey; no unified vehicular uplink implementation
[20]	RAN/RL	Position paper	Highlights generalization and lightweight design needs in RAN RL	Conceptual; limited empirical vehicular validation
[4]	Foundational	Tabular Q-Learning	Simple; convergence guarantees under mild conditions	Overestimation bias due to max operator
[5]	Foundational	Double Q-Learning	Reduces overestimation via decoupled selection/evaluation	Still tabular; scalability requires careful design
[6]	Foundational	UCB exploration	Tight regret in bandits; optimism principle	Needs adaptation beyond bandits for MDPs
[7]	Foundational (tabular MDPs)	UCB-Q Learning	Provable sample efficiency in tabular MDPs	Assumptions/schedules differ from practical vehicular settings

**Table 2:** Comparison of Q-Learning variants for V-IoT spectrum management.

Algorithm	Exploration Strategy	Update Rule	Distinctive Features
Q-Learning (baseline)	ϵ-greedy with decaying ϵ	$Q\left(s,a\right)\leftarrow Q\left(s,a\right)+\alpha \left[r+\gamma \max_{a^{\prime }}Q\left(s^{\prime },a^{\prime }\right)-Q\left(s,a\right)\right]$	Simple adaptive learning but prone to overestimation bias.
Double Q-Learning	ϵ-greedy with decaying ϵ	Two *Q*-tables ($Q^{A},Q^{B}$) are updated alternately using decoupled action selection and evaluation	Reduces overestimation and improves value stability.
Proposed—VPADQ-C	ϵ-greedy with SRP modulation (Equation (9))	DoubleQ update with priority-weighted rewards: $Q^{X}\left(s,a\right)\leftarrow Q^{X}\left(s,a\right)+\alpha \left[\alpha_{k}r+\gamma Q^{Y}\left(s^{\prime },\arg\max_{a^{\prime }}Q^{X}\left(s^{\prime },a^{\prime }\right)\right)-Q^{X}\left(s,a\right)\right]$	Incorporates traffic-class priority into value updates and enforces CMDP safety via dual variables.
Proposed—Q-UCB	Contextual UCB: $\arg\max_{a}\;\left[Q\left(s,a\right)+\beta \;\sigma_{k,t}\left(1-{\hat{\rho }}_{k,t}\right)\sqrt{2\log N_{k,t}/\left(1+n_{a,k,t}\right)}\right]$	Standard Q-Learning update	Improves exploration by balancing exploitation with context-aware statistical confidence; faster convergence in dynamic vehicular environments.

**Table 3:** Simulation configuration parameters.

Parameter	Value/Description
Episodes	1500
Steps per episode	150
Number of devices	6 to 60 (step 6)
Spectrum	100 MHz, *C* orthogonal channels
Slot duration	1 ms
Traffic model	Poisson/Gamma–Poisson arrivals (safety, control, infotainment)
Channel model	$h_{i}=\sqrt{G_{0}d_{i}^{-\alpha }}g_{i}$; log-normal shadowing, Rayleigh fading, Gaussian interference
Spectral efficiency	$\log_{2}\left(1+\mathrm{SINR}\right)$, capped at 6 bits/s/Hz
SINR range	[−20,40] dB (with μ=12 dB, σ=6 dB)
Metrics	Throughput, delay, blocking, utilization, fairness, energy efficiency
Complexity	O(1) per step; $O(N_{\mathrm{devices}}^{2})$ overall

## Data Availability

The data presented in this study are available on request from the corresponding author due to our ongoing project.
